# Feasibility of segmental total body irradiation (SegTBI) using a 1.5T MR‐linac

**DOI:** 10.1002/acm2.70192

**Published:** 2025-07-15

**Authors:** Matthew Manhin Cheung, Ashley Chi Kin Cheng, Louis Lee

**Affiliations:** ^1^ Medical Physics Division Department of Medical Innovation & Technology CUHK Medical Centre Hong Kong SAR China; ^2^ CUHK Medical Centre Hong Kong SAR China; ^3^ Department of Clinical Oncology Faculty of Medicine CUHK Hong Kong SAR China; ^4^ Department of Medicine and Therapeutics Faculty of Medicine CUHK Hong Kong SAR China

**Keywords:** dose feathering, MR Linac, total body irradiation, unity

## Abstract

**Objective:**

To evaluate the technical feasibility of using the elekta unity magnetic resonance linac (MRL) as a backup for tomotherapy in total body irradiation (TBI) treatment.

**Methods:**

A single pediatric patient's TBI treatment with a prescription of 12 Gy in six fractions was retrospectively re‐planned using a multiple‐isocentre approach with dose feathering on the MRL system. An additional plan was created and delivered to an anthropomorphic phantom containing OSLDs. The study investigated the MRL system's limitations and capabilities.

**Results:**

The plan sum of the nine segments not only met the dosimetric criteria of planning targets but also demonstrated the MRL system's capabilities by keeping the mean lung dose below 8 Gy and the mean kidney dose below 10 Gy. The electron streaming effect was observed. Treatment plan verification using ArcCHECK measurements with a global 3%/2 mm gamma analysis had a pass rate greater than 95% for all segments. In 28 out of the 30 OSLDs in brain, bone, and soft tissues, the deviation of the measurement from the reported TPS dose is within ±5%. A much larger deviation was observed in the lung tissues. Segmental TBI using the MRL was a viable option for TBI treatment.

**Significance:**

This study demonstrates the technical feasibility of MRL for TBI by offering dose modulation and imaging capabilities. The MRL can serve as a backup despite longer planning and treatment times. The potential for future workflow optimizations could enhance its practicality. This research improves flexibility in treatment planning and delivery for TBI patients.

## 1 INTRODUCTION

### 1.1 Total body irradiation

Total body irradiation (TBI) can be used as a radiotherapeutic technique to prepare for hematopoietic stem cell transplant.^1^ Conventional methods used extended source‐to‐skin distance (SSD) and large field size to achieve the comprehensive coverage required for TBI. However, such an approach compromises dose uniformity and only allows limited dose modulation. Recent surveys suggested that most centers worldwide still use an extended SSD technique with some field‐in‐field techniques to perform TBI with improved dose homogeneity.^2^ Previous studies^3^, ^4^, ^5^, ^6^, ^7^ proposed volumetric modulated arc therapy (VMAT) with multiple isocentres could improve organ sparing and plan quality in TBI, as well as eliminate the use of compensators and blocks. The intensity modulation combines technological advancements in beam delivery and imaging guidance.

### 1.2 TBI using tomotherapy

Tomotherapy delivers a helical pattern, allowing for highly conformal dose distribution and improved dose homogeneity across the target volume. The prolonged treatment length, continuous delivery, and precise modulation of the radiation beam make tomotherapy one of the ideal therapeutic options for TBI.^8^ Our institution has one Accuray Radixact and one 1.5 T Elekta Unity MRL. While helical tomotherapy was naturally selected to treat TBI patients, it is intuitive to consider using the MRL as a backup machine during tomotherapy machine failure or downtime because the risk of treatment interruptions should be minimized to avoid compromising the transplant outcome.^9^ One study^10^ demonstrated that VMAT using Varian Truebeam STx could be used as a backup to the tomotherapy machine, and there had been one actual case irradiated with both modalities due to a device failure of the tomotherapy machine.

### 1.3 Elekta unity overview

The Elekta Unity MRL integrates a 1.5T high‐field MRI scanner with a 7 MV flattening filter‐free (FFF) LINAC system. The Monaco treatment planning system (TPS) takes into account the effect of the magnetic field. The excellent soft tissue contrast in 3D MRI enables daily adaptive treatment,^11^ allowing for real‐time tumor visualization and treatment gating.^12^ The system has a source‐to‐axis distance of 143.5 cm and a maximum field size of 57.4 cm × 22 cm. There are 160 multileaf collimator (MLC) leaves with a nominal pitch of 0.7175 cm in the IEC‐y direction and diaphragms in the IEC‐x direction. The couch can be moved in the longitudinal direction only, and the bore diameter is 70 cm.

### 1.4 Limitations of the MRL for TBI

Several limitations render the MRL less than ideal for TBI treatment. The major one is that the maximum field size along the IEC‐y (cranial‐caudal) direction is only 22 cm, and multiple isocenters are, therefore, mandatory for a TBI treatment. It was reported that a dual‐isocentre approach in the MRL could be applied to treating cervical cancer^13^ by creating a dose gradient between the field junctions.

It is mandatory to acquire a 3D MR acquisition in the Unity MRL workflow for fractional plan adaptation, which significantly increases the total treatment time in addition to the beam delivery. The dose rate of 425 MU/min is inherent to the Unity MRL, which may be suboptimal as lower dose rates are desirable in the lung regions.

The collimator is not rotatable in the MRL. However, it is less of a problem in this particular clinical application of TBI, as using MLC along the IEC‐y direction may be beneficial in sparing the lungs.^14^, ^15^

Compared with conventional linacs, where there is a maximum over‐travel limit for the MLC, the MLC of the MRL can move in the full range in the MRL. For the ring‐mounted Varian Halcyon and Ethos system, the MLC leaves can travel a longer distance to cover the entire field size of 28 cm.^16^ A previous feasibility study using Halcyon demonstrated that seven segments and 13 isocentres could treat a patient with a body length of 162 cm.^17^

The only available dose calculation algorithm for the MRL system is Monte Carlo‐based, and the dose calculation time could be longer than other convolution‐based algorithms. However, it could be a more accurate representation of the dose deposition in tissue inhomogeneities.

Only flattening filter‐free beams are available, so intensity modulation is inevitable in the MRL and a simple parallel opposing field approach is not applicable. In addition, patients with MR contraindications could not be treated and MR safety should be assessed for implants or foreign metallic objects. The immobilization devices should be compatible with the MRL system, causing no harm to the patient and producing minimal to no imaging artifacts. All the immobilization devices at our institution are MR‐safe or conditional to enable machine interchangeability after the acquisition of planning CT.

### 1.5 Features of the MRL for TBI

Despite the challenges and limitations of the MRL system, there are still a few advantages and unique characteristics of using the Elekta Unity machine to treat TBI patients. First, image guidance is available for every segment before beam delivery. The image resolution in MRL can be adjusted with the Monaco 5.51.11 (Elekta, Crawley, UK) and MRI control software Marlin 5.7.1 (Philips, Netherlands). Compared with other clinical applications like Stereotactic Body Radiation Therapy of different disease sites in which the MRI scan can take several minutes, a faster pre‐treatment scan with coarser resolution is sufficient for TBI. The 3D MRI acquisition with 2–5 mm resolution may be a practical choice, and a scan time of less than 1 min per isocentre can be achieved. The actual MR scanning time depends on the desired spatial resolution, contrast and signal‐to‐noise ratio.

The couch position of the MRL that can be moved to the isocentre is 174 cm (Unity couch index position 43.5), and such an extended couch travel capacity allows most of the patients to be treated in a head‐first‐only orientation and a change to a feet‐first orientation to complete the whole length of TBI is unnecessary. The couch sagging is also unlikely to be a problem as the entire length of the couch is supported inside the bore for every segment, in addition, image guidance is always available to check the final position.

The electron return effect due to the presence of the magnetic field^18^ might be beneficial in lung‐sparing, which is essential in TBI treatment. The air‐tissue interfaces in the lungs could also cause a build‐up of dose in the chest wall, and increased dose coverage of the ribs is desired.^19^

Real‐time imaging is also available in multiple 2D orthogonal planes to ensure timely beam interruption for sudden movements during treatment. The future upgrade of the system will also enable automatic beam gating.^12^

This study addresses the question of whether segTBI in MRL is technically feasible, even with the limitations listed above.

## 2 METHODS

### 2.1 Pediatric patient plan

This retrospective study was approved by the institutional Clinical Research Ethics Committee. A patient with a height of 137 cm received TBI (12 Gy in 6 fractions twice daily) treatment with a tomotherapy machine at our institution. The CT images were retrospectively planned for multiple isocentre TBI in the MRL system. The patient's knees were bent so that a single plan of length 125 cm could be used to cover the entire body length in the tomotherapy machine. The patient was immobilized using a large Klarity vacuum bag (200 cm × 100 cm) with 120‐liter fill. A large thermoplastic mask was used to fix the head and neck region.

**FIGURE 1:**
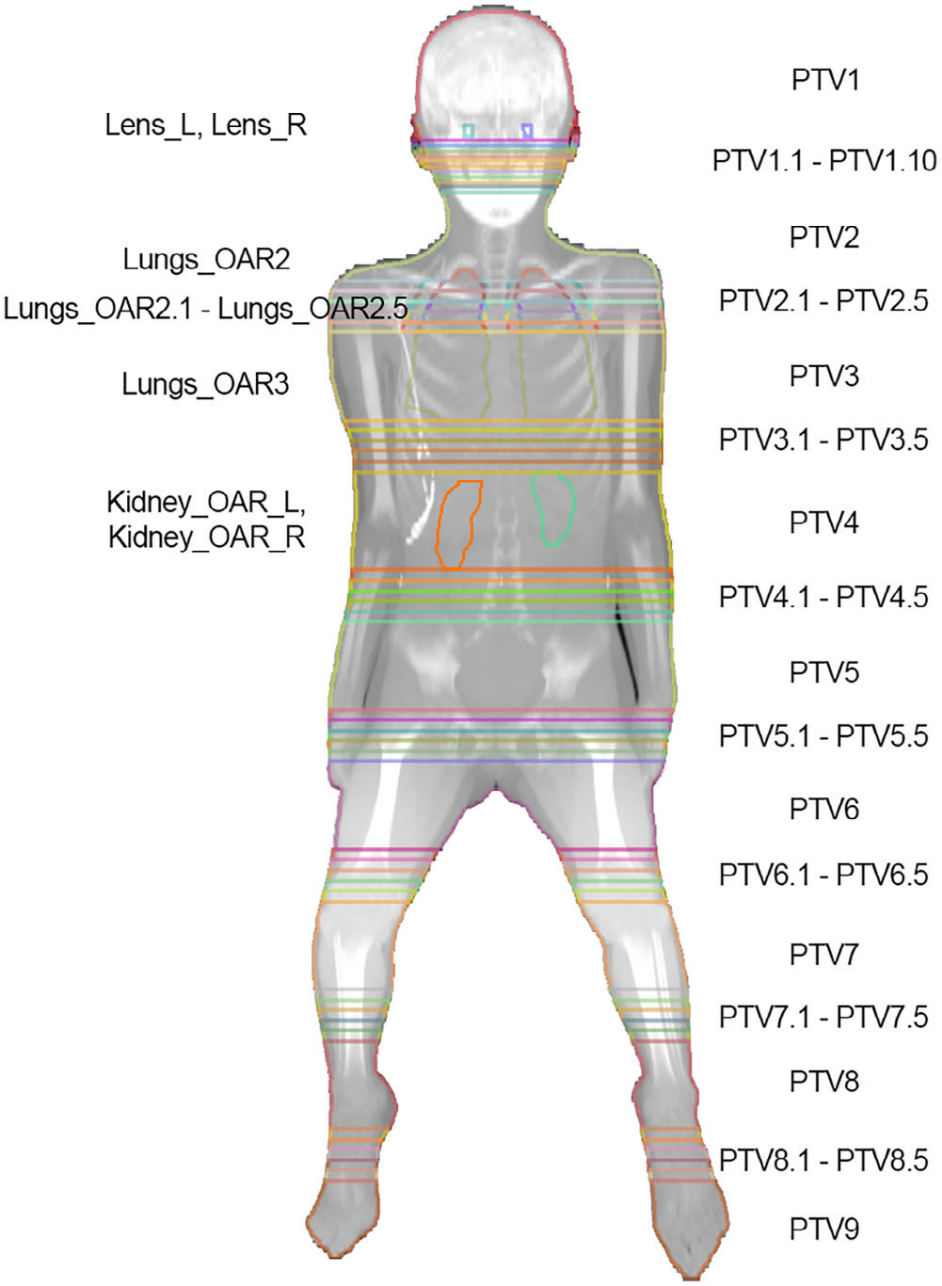
Optimization structures overlaid on the maximum intensity projection of the CT coronal images. Nine segments are created for optimization. Thinner overlapping slices are shown between the first two segments in the head and neck region. The OARs, including lungs, lenses, and kidneys, are outlined and segmented if needed. The body contour is cropped 3 mm as the PTV.

The planning CT was acquired using a Siemens Somatom Force scanner with a 5 mm slice thickness, 80 kV_p_, CTDI_vol_ = 3.87 mGy, and DLP = 532.4 mGy cm. Direct density^20^ was used to reconstruct the images. The TPS, Monaco (Elekta, Crawley, UK), was used to create the segmental plans in this study. As shown in Figure 1, the CT was segmented into slabs of 18 cm, with 5 cm of overlap between consecutive segments, and the central 8 cm without any overlap, using MIM Maestro 7.4.2.

The kidneys and lung structures were contracted 5 mm and referred to as Kidneys_OAR and Lungs_OAR, respectively, from the original structures for optimization and evaluation. The PTV was cropped 3 mm from the external body contour. The PTV Body_net structure is the PTV subtracting the lenses, kidneys and lungs for evaluation. Every PTV or OAR within two slices (1 cm) in the overlapping region was contoured, except that in the head and neck region, each 5 mm slice was contoured as shown in Figure 1. The structures PTV1.1–PTV1.10 were also grouped into a structure PTV1_inf for bias dose optimization. The same grouping was also done for each segment.

During the optimization process, the PTV and OAR in the planning and adjacent segments were assigned optimization constraints. There were 15–17 beams in each segment. Optimization with dose gradients as base dose forced the subsequent segments to deliver a dose distribution with a gradual fall‐off of about 2 Gy or 16% of the prescribed dose per cm in the junction regions. In order to speed up the optimization process, the plans for each segment were first optimized with around 80 CT slices with a bias dose planning function in Monaco using a 5 mm grid size and 1% statistical uncertainty. The planning parameters controlling the details for control points were adjusted manually during the plan optimization process to minimize the treatment delivery time while ensuring the dose criteria could be met. The optimization constraints and sequencing parameters for optimization are shown in Figure S1. The tissue heterogeneity in the lungs led to relatively tight weighting and sequencing parameters in segment 2. The fluence smoothing was set to medium to high for segments other than 1–3 to minimize the treatment delivery time. The plans were then recalculated in the whole‐body CT images with a 3 mm grid size and 1% statistical uncertainty. The MU rescales were done in each plan if needed.

The option “autoflash” in Monaco was set to 1 cm to control MLC shaping, improve dose distribution robustness against possible setup errors, and account for intra‐fractional motion. A fill of 0.01 g/cm^3^ relative electron density was used to ensure that all immobilization devices outside the external body were included during optimization.^21^ The target‐volume prescription goals and OAR constraints are shown in Table 1.

**TABLE 1:** Dosimetric criterion of the target and OARs for the pediatric patient plan.

		Value		
Structure	Dosimetric criterion	Plan sum	Shifted seg 2 by 5 mm	Shifted seg 2 by 1 cm
PTV body_net	V_11.4_Gy > 95%	99.17%	99.08%	96.58%
	V_10.8_Gy > 98%	99.79%	99.83%	98.34%
	V_14.4_Gy < 1%	0.00%	0.26%	1.52%
	V_13.2_cGy < 5%	4.31%	6.59%	8.50%
Lungs_OAR	Dmean < 8 Gy	7.85 Gy	8.06 Gy	8.33 Gy
Kidney OAR_L	Dmean < 10 Gy	9.74 Gy	9.74 Gy	9.74 Gy
Kidney OAR_R	Dmean < 10 Gy	9.90 Gy	9.90 Gy	9.90 Gy
Lens_L	Dmax < 11.4 Gy	11.35 Gy	11.22 Gy	11.11 Gy
Lens_R	Dmax < 11.4 Gy	11.33 Gy	11.02 Gy	10.90 Gy

Values that could not meet the dosimetric criterion were in red.

The plans were mapped to Sun Nuclear ArcCheck and delivered in quality assurance (QA) mode to verify the plan's specific QA. The tolerance and action limits of the gamma passing rate follow the recommendation of AAPM TG Report 218.^22^

To evaluate the dosimetric impact of a potential shift in the cranial‐caudal direction, segment 2 was shifted by 5 mm and 1 cm in the caudal direction, and the dose was re‐calculated.

### 2.2 Dose verification using anthropomorphic phantom and OSLD

An anthropomorphic adult female phantom (ATOM, Sun Nuclear) consists of 38 horizontal sections, each 25 mm thick, and holes drilled 14 mm in diameter for insertion of optically stimulated luminescent dosimeters (OSLDs). The locations of the holes represent the positions of different organs. The organs were contoured to mimic the clinical scenario, and a TBI plan with seven isocentres was created with the same optimization constraints listed in Table 1.

The OSLD system (RadPro International System GmbH) comprises an OSL reader, an eraser, and OSLDs made from beryllium oxide, with a 4.65 mm × 4.65 mm × 0.5 mm sensitive element encapsulated with a holder. The OSLDs were calibrated and calibration factors for each OSLD were obtained by irradiating 2 Gy using the 6 MV FFF Tomotherapy machine. The beam quality correction factor was taken as unity in our internal tests, which aligns with the findings of other studies.^23^, ^24^, ^25^ To minimize the presence of an air gap, a 23‐gauge syringe was used to fill the cavity with water as much as possible.

**FIGURE 2:**
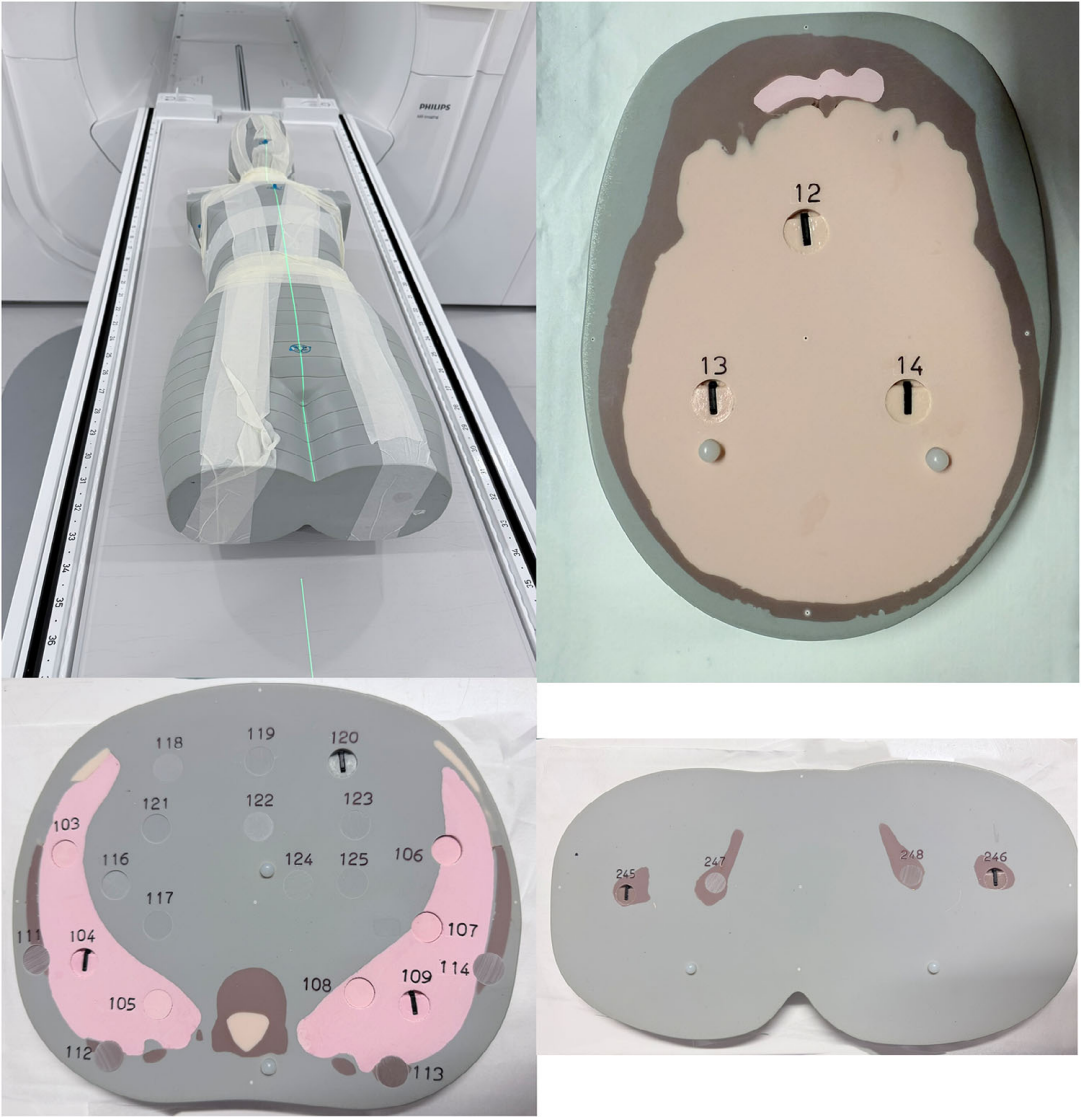
The anthropomorphic phantom with through‐holes drilled for the insertion of plugs containing OSLDs. Three representative horizontal sections for the head, thorax, and pelvis regions are shown with insertion of plugs mimicking brain tissue (yellow), lungs (pink), soft tissue (grey) and bone tissues (brown).

The TBI plan was recalculated using the CT Image set reconstructed with a 2 mm slice thickness and 40 OSLDs (16 in soft tissue, 10 in lung, eight in brain, and six in bone tissue plugs) inserted throughout the body. Representative sections are shown in Figure 2. The dose calculation resolution was set to 2 mm, and the uncertainty was 0.5% per calculation.

As the phantom is not visible in MRI, only the MR markers were used to aid in the positioning of the phantom on the Unity. Bottles of solutions were placed next to the phantom to provide an adequate signal during each MR acquisition, and these bottles were removed before irradiation. Plan adaptation was not performed and one fraction of the original segments of the reference plan were delivered.

The OSLDs were dried and placed in a drying cabinet for 3 h before reading. The data were then compared with the mean dose of the OSLD ROIs reported by the TPS.

## 3 RESULTS

### 3.1 Pediatric patient plan

**FIGURE 3:**
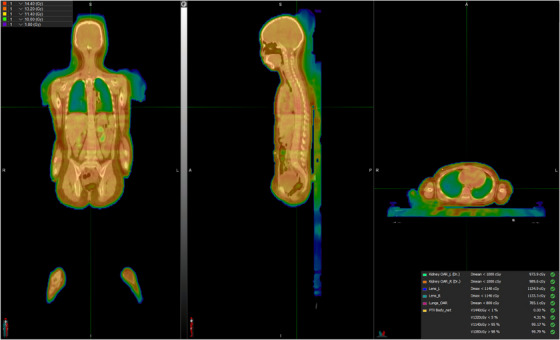
Coronal, sagittal, and axial plane for the dose distribution of the plan sum of the patient plan.

The pediatric patient plans were generated using nine segments, and the dose distribution is shown in Figure 3. The volume coverage of 95% dose was higher than 99%, and all the dosimetric criteria were met, as shown in Table 1. The use of bias dose planning was not allowed to be approved in the current system, and the plans had to be saved as a template and imported again for re‐calculation before approval.

Table 2 lists the plan details and the QA results. The gamma passing rates were within the tolerance limits (95%) with a global 3%/2 mm, and a 10% threshold for gamma analysis. The gamma passing rate did not demonstrate a decreasing trend with the number of segments or MU. A total of 5291.6 MU in 861 segments was delivered with a maximum dose rate of 425 MU min^−1^. The total beam‐on time needed for delivery was 78.6 min. Unsurprisingly, the intensity modulation was mainly found in the third segment, where the majority of the lungs fell within.

**TABLE 2:** Summary of the plan delivery and the plan‐specific QA results by ArcCheck measurement.

Segment	MU	Number of segments	Beam‐on time (min)	ϒ‐passing rate (3%, 2 mm, TH 10%)
1	393.5	73	6.3	97.5%
2	460.89	81	7.1	99.7%
3	956.63	164	15.5	95.1%
4	678.32	115	10.0	97.7%
5	298.59	59	5.2	95.3%
6	679.83	125	11.2	96.8%
7	980.12	95	10.7	95.3%
8	425.01	71	6.7	99.6%
9	418.84	78	5.9	95.8%

**FIGURE 4:**
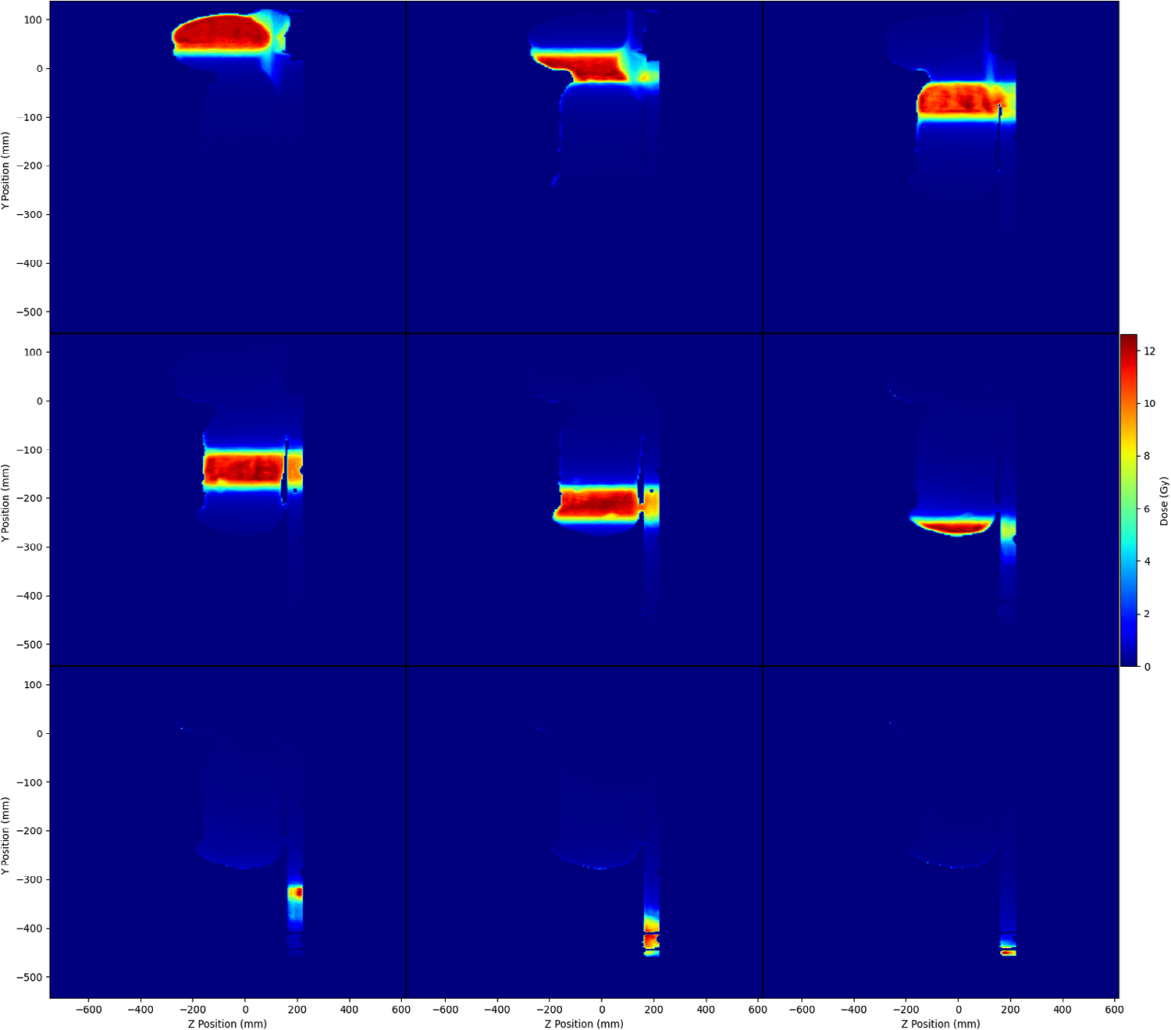
Sagittal dose plane of the mid‐line of individual segments of the patient plan. Apart from the dose feathering effects, the increase in skin dose can also be observed in the out‐of‐field regions in individual segments.

**FIGURE 5:**
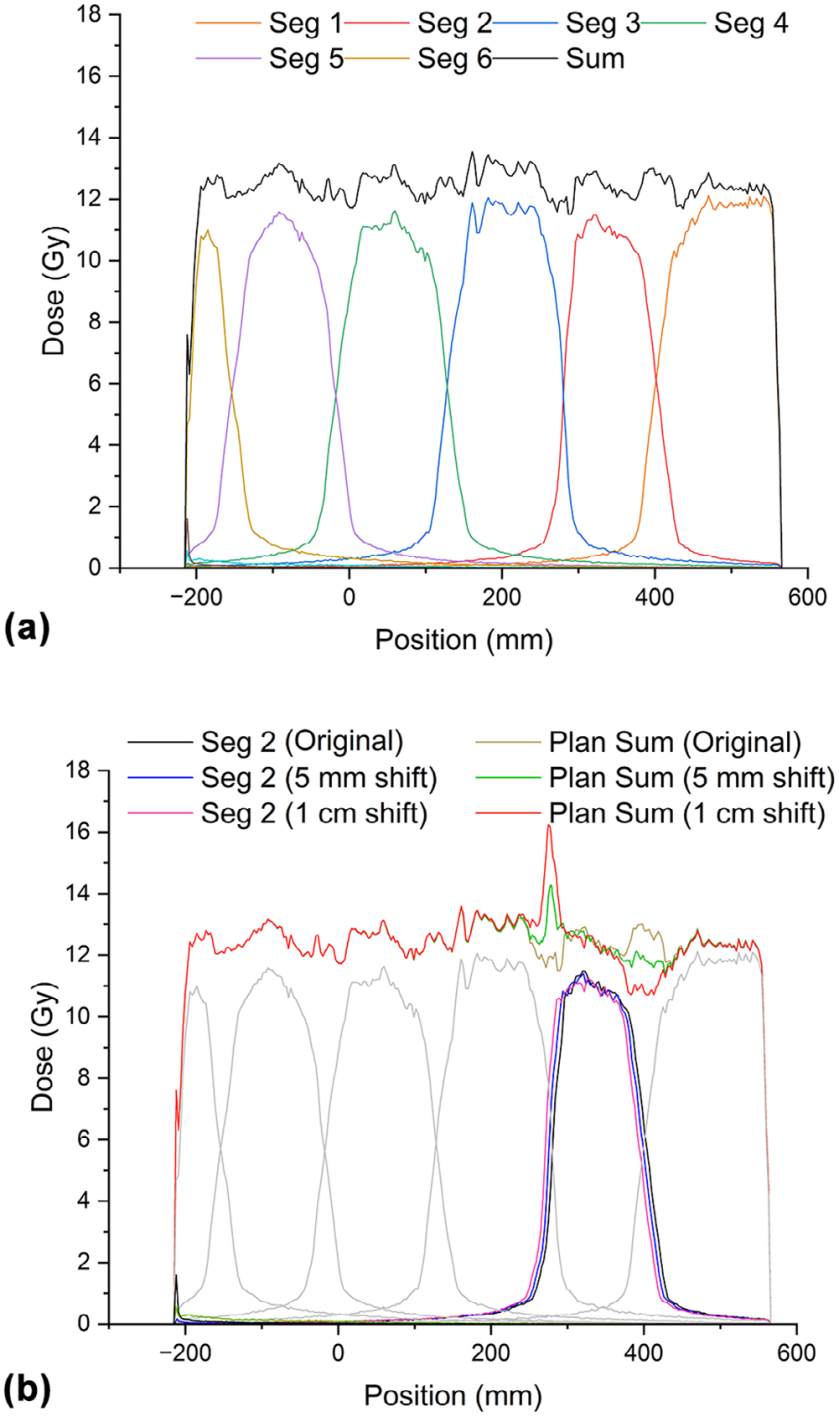
(a) Dose profile for individual segments and the plan sum along y‐direction at the mid‐position of the patient. The overlapping dose gradients are about 5 cm in individual segments. (b) The dose profiles of segment 2 were re‐calculated after drifting 5 mm and 1 cm in the caudal direction. Segments 7–9 are not shown in the diagram as there is no dose in the mid‐sagittal plane.

The mid‐sagittal dose planes are displayed in Figure 4. The electron streaming effect (ESE) leading to increased skin dose in the out‐of‐field region was notable. An out‐of‐field dose of approximately 1.5 Gy was observed at the surface of the adjacent segments. The bias dose optimization took such effect into account during optimization. Irradiation in the individual segments could lead to higher skin dose in adjacent segments. The 1D dose profiles at the mid‐position of the patient showing the dose contributions from the individual segments and the total plan sum along the cranial‐caudal direction are plotted in Figure 5a. The dose gradients along different segments form a reasonably uniform total dose. Similar plots are displayed in Figure 5b to demonstrate the effect of shifting segment 2 by 5 mm and 1 cm caudally. The PTV coverage at 11.4 Gy was relatively immune to such a single‐segment shift. The D_105%_ (13.2 Gy), however, exceeded the constraints of 5% volume even with a 5 mm shift. The mean lung dose also marginally failed to meet the constraint of 8 Gy, with a 5 mm shift, and could reach 8.33 Gy after a 1 cm shift. The hotspot, as defined by a dose of 14.4 Gy, exceeded a 1% volume, as the overlapping region was 1 cm.

### 3.2 Dose verification using anthropomorphic phantom and OSLD

The CT image of the phantom is displayed in Figure S2, and the created TBI plan is shown in Figure S3. The location and individual dose comparison of the 40 OSLDs are listed in Table S1, and the dosimetric verification results are summarized in Table 3. In 28 out of the 30 OSLDs in brain, bone, and soft tissues, the deviation of the measurement from the reported TPS dose lay within ±5%. A significant deviation up to 11.4% was observed in the lung tissues.

**TABLE 3:** Dosimetric results: comparison between OSLD and TPS.

Tissue types	Number of OSLDs	OSLD (Gy)	TPS (Gy)	Difference (%)	
		(Mean ± SD)	(Mean ± SD)	(Mean ± SD)	(Range)
Soft Tissues	16	2.12 ± 0.15	2.10 ± 0.16	−1.2 ± 2.62	−5.23, 3.29
Brain	8	2.16 ± 0.04	2.12 ± 0.03	1.86 ± 1.84	−0.54, 5.95
Bone	6	2.10 ± 0.09	2.11 ± 0.13	−0.29 ± 3.16	−4.89, 4.12
Lungs	10	1.20 ± 0.16	1.30 ± 0.16	−7.58 ± 3.54	−11.38, 0.09

## 4 DISCUSSION

### 4.1 Planning and treatment time

One of the main limitations of using the MRL for TBI patients is the lengthy time for planning and treatment. However, efficiency can be significantly increased after the standard template is created. Automated processes such as scripting^5^ or deep learning methods are warranted for faster planning. Auto feathering algorithm for cranial‐spinal irradiation treatment was demonstrated with the VMAT technique.^26^ Similar processes should be implemented in the MRL to overcome the limitation of maximum field size. The total on‐couch time for patients was aimed to be approximately 2 h in this study. It should be achievable with optimization in the imaging sequence, limited segment numbers in plan optimization, and a streamlined workflow during treatment. The contoured PTV and OARs were relatively simple in TBI, and the online adaptive feature of updated contouring in MR‐Linac would likely not be needed. The adapt‐to‐position (ATP) approach of adjusting the plan to correct for shifts in patient position would be sufficient. The ATP approach still requires optimization and re‐calculation. The use of a 3 mm dose grid in this study may be relaxed to 5 mm, which is generally accepted for multiple‐isocentre VMAT TBI.^21^ Using auto‐flash may also increase the chance that the original plan segment may be acceptable to the oncologists.

### 4.2 Electron streaming effect

For beams incident on oblique tissue‐air interfaces, secondary electrons entering the air will have trajectories following the direction of the main magnetic field, which is the cranial‐caudal direction.^27^ This is referred to as the ESE effect and contributes to out‐of‐field dose. The ESE effect was prominent in Figure 4 and should be addressed in real clinical cases. Although the bias dose planning in the TPS considered such an effect, the prediction may not be accurate.^28^ Shielding should be implemented, and a bolus of 1 cm was shown to minimize the radiation dose effectively.^29^ Apart from regions like the chin, breast, and arms, any tilting of the patient's position should be considered. For example, for the position in the current study in which the patient's knees were bent, the ESE could not be overlooked. The regions that are not flat and not being treated are recommended to be covered by bolus.

### 4.3 Dose feathering

The gradients could be formed in most regions along the desired caudal cranial directions. In the initial attempts in planning the pediatric patient plan, however, it was found that electron density inhomogeneities due to air and bones in the head and neck region made it challenging for the optimizer to form a gradient along a single direction only. Setting minimum and maximum dose constraints to a slab often led to heterogeneous in‐plane dose distribution. To better control the cross‐plane dose gradients, slices were created in a 5 mm step instead of 1 cm in the overlapping region between the first and second segments. The length of the feathering zone was a balance between the vulnerability to a possible shift in that direction and the number of isocenters to be used. Segment 2 was chosen to shift towards the caudal direction to mainly evaluate how the dose coverage and mean lung dose would be affected. The overlapping length was designed to be 5 cm in this study, and the optimization tended to increase the dose coverage as long as the OAR constraints were not violated. Even with a 1 cm shift for segment 2 in this study, the mean lung dose differed by less than 5% in Table 1. In this planning study, a nearly linear dose fall‐off of 2 Gy per cm was achieved in the individual segments, facilitating an estimation of the dose in the hot and cold regions. Such simplification heavily depends on tissue homogeneity, and in particular, predicting lung doses would be challenging due to the presence of the magnetic field. Depending on the actual clinical scenarios, the length of the overlapping region could be adjusted. This study demonstrated that such an effect could be minimized, and the desired dose uniformity was achievable in optimal segmentation. It could potentially be applied in other sites where the required treatment length exceeds the maximum field size.

### 4.4 Patient eligibility for MRL‐based TBI

MR assessment must be performed before a patient can be treated in the MRL. Although the couch can move up to 174 cm, the patient height would determine the number of isocentres required. As the maximum field width in the IEC‐x direction is 57.4 cm, the width of the patient would not significantly affect the treatment time. The placement of the MR coil only allows about 35 cm separation in the IEC‐z direction, limiting the extent of knee bending and the thickness of the vacuum bag. A Unity clearance tool, as shown in Figure S4, having the same clearance as the MR coil can be used during CT simulation to ensure the immobilization device would not exceed the physical allowable dimension.

### 4.5 Dose verification using anthropomorphic phantom and OSLD

Dose verification in the presence of magnetic field has been challenging as the presence of small submillimeter air gaps around the dosimeters could lead to a significant dose difference.^30^, ^31^, ^32^ The effect of Lorentz force induced dose changes strongly depends on density differences of the materials.^33^ The response of the detectors also depends on the beam orientation with respect to the tissue‐air interface.^25^, ^30^ With water added to minimize air gaps, the measurements were shown in previous reports to significantly improve the uncertainty.^23^, ^34^ In this study, the OSLDs are orientated along the sagittal plane and the angular dependence of OSLD response was approximated to be unity as the gantry angles were distributed evenly. In our results, apart from the lungs, the OSLD measurements reasonably agreed with the TPS reported dose, demonstrating the feasibility of dose verification of total body treatment in MRL. The detector‐lung interface, however, introduced a large uncertainty to the dose measurement and further work is warranted to examine a reliable in vivo dose verification in the lung regions.

### 4.6 Limitations

One limitation of this study is that only one subject was involved, similar to previous studies.^17^, ^35^ In particular, the variability of patient size and tissue inhomogeneities could potentially impact the feasibility and effectiveness of the proposed approach. The primary purpose of this study was to investigate the feasibility and explore the possible challenges and considerations of employing the MRL. The concepts and practices can only be generalized to clinical cases with further investigation of differences in patient anatomy.

The current study is a planning study, and the plan‐specific QA of individual segments was performed using ArcCHECK. Further investigation on dosimetric verification of the plan sum is warranted in future studies.

### 4.7 MRL as a backup machine for TBI

The MRL is capable of performing real‐time imaging and ensuring an accurate dose delivery while providing dose modulation in a TBI treatment. Nevertheless, the duration was still considerably longer than other modalities, including tomotherapy, and therefore, the MRL was used only as a backup machine in our institution practically. Also, a previous report^36^ showed that while both VMAT and tomotherapy could produce acceptable quality, the dose uniformity using tomotherapy could be superior. It should be noted that no significant clinical differences were detected in acute and subacute toxicity between the use of Tomotherapy and VMAT groups in that study, and no late toxicity was observed in that study, suggesting that using IMRT may provide a similar clinically safe backup plan if the OAR dose constraints could be achieved.

## 5 CONCLUSION

This study has demonstrated that SegTBI on the MRL is technically feasible. Implementation of automatic processing may shorten the planning and treatment time in the future.

## AUTHOR CONTRIBUTIONS

The authors confirm their contribution to the paper as follows: Study conception and design: Ashley Chi Kin Cheng, Louis Lee. Data collection: Matthew Manhin Cheung. Analysis and interpretation of results: Matthew Manhin Cheung, Ashley Chi Kin Cheng, Louis Lee. Draft manuscript preparation: Matthew Manhin Cheung, Louis Lee. All authors reviewed the results and approved the final version of the manuscript.

## CONFLICT OF INTEREST STATEMENT

The authors declare no conflicts of interest.

## Supporting information



Supporting Information
