# Supplementary material for: Feasibility of segmental total body irradiation (SegTBI) using a 1.5T MR‐linac
Source: J Appl Clin Med Phys. 2025 Jul 15;26(7):e70192. doi: 10.1002/acm2.70192 (PMC12263254; doi:10.1002/acm2.70192)
Supplement: Supplementary file 1 — Supporting Information [file ACM2-26-e70192-s001.docx]

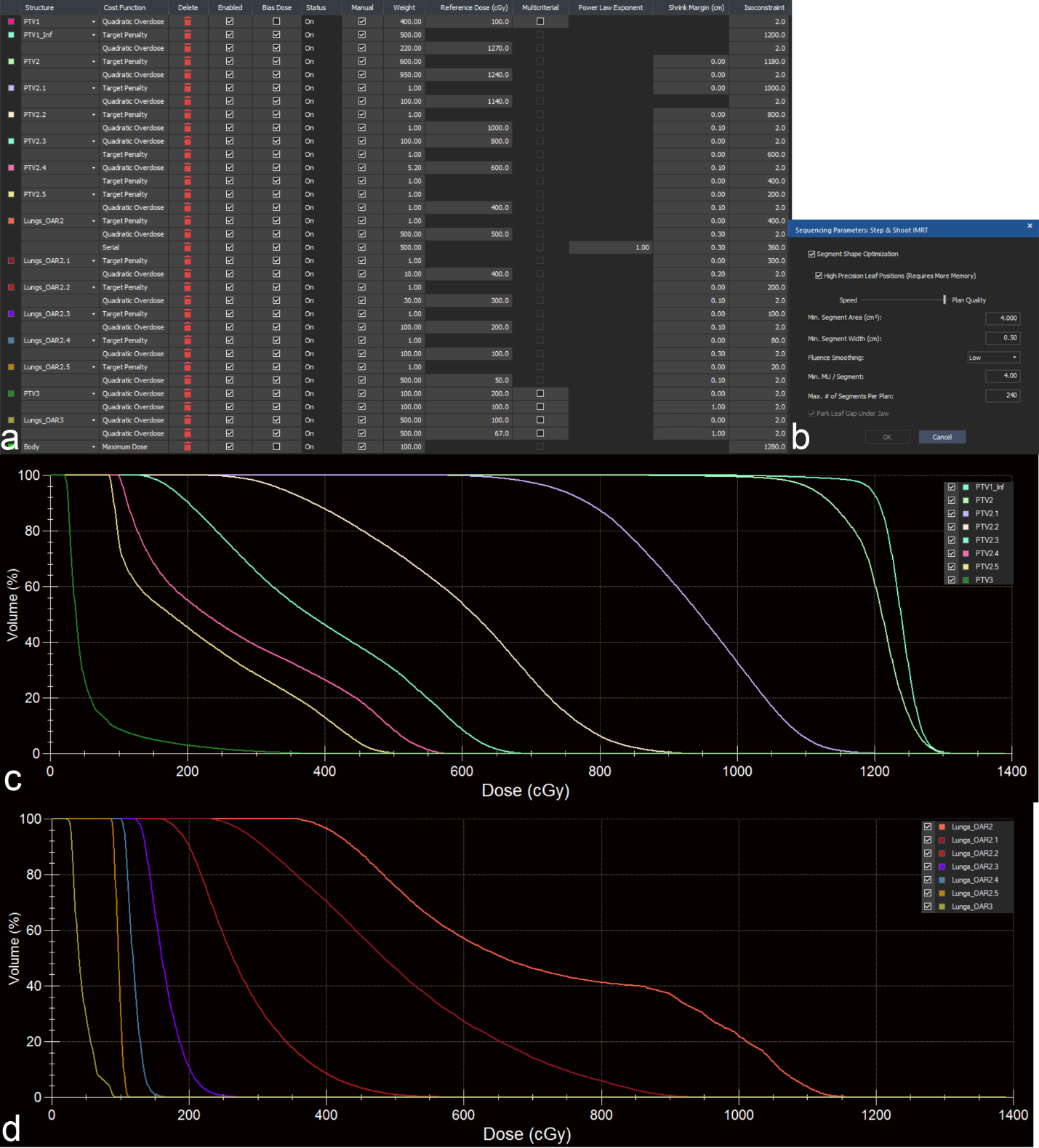


**Supplementary Figure 1** Optimization of segment 2 of the pediatric patient plan. a) Optimization constraints used. b) Sequencing parameters used. c) DVH of the PTV and d) lungs including the dose of segment 1.


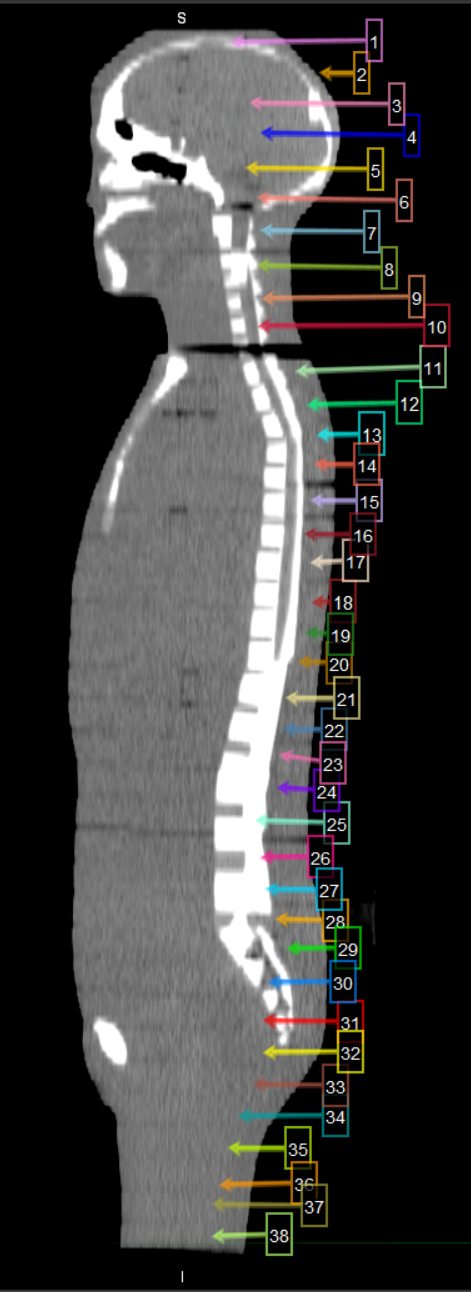


**Supplementary Figure 2** The anthropomorphic phantom consists of 38 slices, each with 25 mm thick.


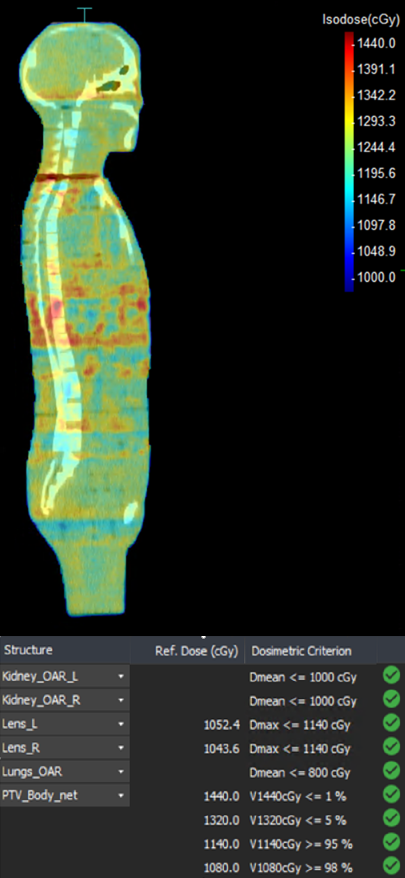


**Supplementary Figure 3** The TBI plan of the anthropomorphic phantom.


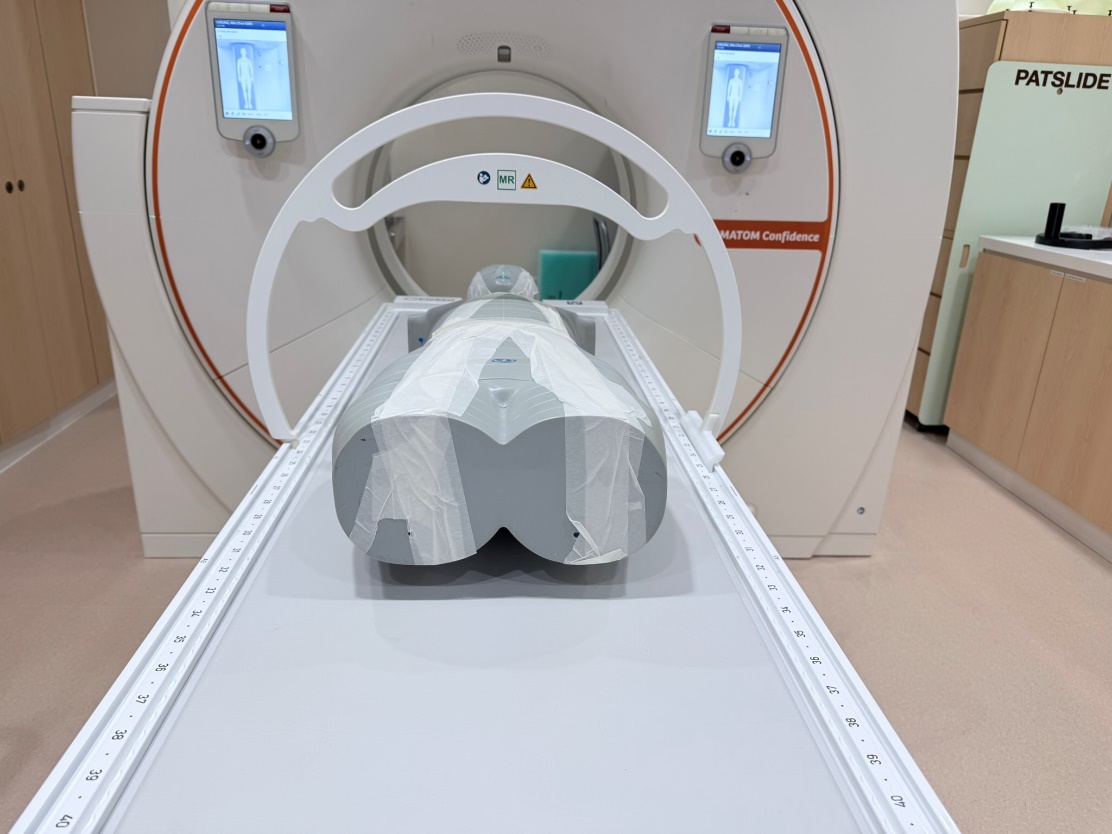


**Supplementary Figure 4** A Unity clearance tool having the same dimension as the MR coil is used during CT simulation process.

|  | **Organs** | **Section Number** | **Detector Location** | **OSLD (Gy)** | **TPS (Gy)** | **Difference (%)** |
| --- | --- | --- | --- | --- | --- | --- |
| **Soft Tissues** | Thyroid | 10 | 26 | 2.162 | 2.138 | -1.13% |
|  | Heart | 16 | 83 | 2.183 | 2.244 | 2.79% |
|  | Liver | 20 | 120 | 2.220 | 2.245 | 1.14% |
|  | Spleen | 21 | 127 | 2.200 | 2.272 | 3.29% |
|  | Liver | 22 | 138 | 2.082 | 2.122 | 1.94% |
|  | Stomach |  | 146 | 2.055 | 1.997 | -2.81% |
|  | Gall Bladder | 23 | 153 | 2.200 | 2.115 | -3.88% |
|  | Pancreas | 24 | 164 | 2.077 | 1.995 | -3.98% |
|  | Kidney |  | 167 | 1.982 | 1.878 | -5.23% |
|  |  |  | 169 | 1.667 | 1.632 | -2.14% |
|  | Stomach | 25 | 185 | 2.185 | 2.110 | -3.43% |
|  | Intestine | 28 | 204 | 2.227 | 2.225 | -0.11% |
|  |  | 30 | 214 | 2.219 | 2.166 | -2.39% |
|  | Ovaries | 31 | 223 | 2.229 | 2.184 | -2.00% |
|  | Uterus | 32 | 239 | 2.269 | 2.211 | -2.59% |
|  | Bladder | 33 | 242 | 2.029 | 2.057 | 1.38% |
| **Brain** | Brain | 2 | 1 | 2.142 | 2.112 | 1.41% |
|  |  |  | 2 | 2.193 | 2.070 | 5.95% |
|  |  |  | 3 | 2.150 | 2.110 | 1.90% |
|  |  | 3 | 5 | 2.149 | 2.118 | 1.51% |
|  |  | 4 | 12 | 2.197 | 2.154 | 2.01% |
|  |  |  | 13 | 2.175 | 2.139 | 1.72% |
|  |  |  | 14 | 2.093 | 2.105 | -0.54% |
|  |  | 5 | 15 | 2.202 | 2.182 | 0.91% |
| **Bone** | Sternum | 14 | 59 | 2.152 | 2.109 | 2.02% |
|  | Ribs |  | 61 | 1.980 | 1.902 | 4.12% |
|  |  |  | 64 | 1.985 | 2.030 | -2.26% |
|  | Thoracic Spine | 15 | 70 | 2.165 | 2.168 | -0.12% |
|  | Femura | 34 | 245 | 2.161 | 2.272 | -4.89% |
|  |  |  | 246 | 2.147 | 2.161 | -0.63% |
| **Lungs** | Lungs | 12 | 34 | 1.072 | 1.209 | -11.38% |
|  |  |  | 35 | 1.279 | 1.390 | -7.99% |
|  |  | 14 | 51 | 1.153 | 1.273 | -9.46% |
|  |  |  | 55 | 1.097 | 1.188 | -7.66% |
|  |  | 16 | 74 | 1.514 | 1.579 | -4.07% |
|  |  |  | 79 | 0.956 | 1.075 | -11.11% |
|  |  | 18 | 94 | 1.341 | 1.499 | -10.56% |
|  |  |  | 98 | 1.264 | 1.368 | -7.62% |
|  |  | 20 | 104 | 1.114 | 1.185 | -6.01% |
|  |  |  | 109 | 1.188 | 1.187 | 0.09% |

**Supplementary Table 1** Detailed dosimetric verification of the OSLDs on the treatment plan of the anthropomorphic phantom. The OSLD were distributed across the phantom and the positions are representative of different organs according to the manual of the organ dosimetry phantom.
